# An in silico LLPS perturbation approach in the design of a novel SARS-CoV-2 spike receptor-binding domain inhibitor

**DOI:** 10.1186/s43042-020-00105-0

**Published:** 2020-11-25

**Authors:** Olanrewaju Ayodeji Durojaye, Divine Mensah Sedzro, Talifhani Mushiana, Henrietta Onyinye Uzoeto, Samuel Cosmas, Judith Nnedimkpa Ajima, Glory Omini Ibiang

**Affiliations:** 1 School of Life Sciences, Department of Molecular and Cell Biology, University of Science and Technology of China, Hefei, China; 2 Department of Biochemistry, University of Nigeria, Nsukka, Enugu State Nigeria; 3 Department of Chemical Sciences, Coal City University, Emene, Enugu State Nigeria; 4 School of Chemistry and Material Sciences, Department of Chemistry, University of Science and Technology of China, Hefei, China; 5 Department of Biological Sciences, Coal City University, Emene, Enugu State Nigeria

## Abstract

The reversible process where a homogenous fluid de-mixes into two distinctively separate liquid phases is referred to as LLPS (Liquid-liquid phase separation). The resulting liquid is made up of one dilute phase and one condensed phase. An increasing number of studies have shown that the liquid-liquid phase separation is an important principle that underlies intracellular organization in biological systems, forming liquid condensates without a membrane envelope, otherwise known as MLOs (membraneless organelles). Such organelles include the P bodies, nucleolus and stress granules. Moreover, the regulation of many other biological processes such as signal transduction, chromatin rearrangement and RNA metabolism have been linked to the liquid-liquid phase separation.

**Dear Editor,**

## Background

The reversible process where a homogenous fluid de-mixes into two distinctively separate liquid phases is referred to as LLPS (liquid-liquid phase separation). The resulting liquid is made up of one dilute phase and one condensed phase. An increasing number of studies have shown that the liquid-liquid phase separation is an important principle that underlies intracellular organization in biological systems, forming liquid condensates without a membrane envelope, otherwise known as MLOs (membraneless organelles). Such organelles include the P bodies, nucleolus, and stress granules. Moreover, the regulation of many other biological processes such as signal transduction, chromatin rearrangement, and RNA metabolism has been linked to the liquid-liquid phase separation [1].

A growing number of studies in recent years have focused on the mechanism of phase separation of a variety of biomolecules. Such studies have demonstrated that some proteins, such as the RNA helicase DEAD-Box 4 (DDX4), P granule protein LAF-1, transactive response DNA-binding protein (TDP-43), and the RNA-binding FUS protein, can undergo liquid-liquid phase separation both in vitro and in vivo. The resultant liquid condensates from the liquid-liquid phase separation process generally are deemed as a product of multivalent weak interactions between the numerous interacting motifs in IDRs (intrinsically disordered regions) or multiple folded domains. The LCRs (low complexity regions), which are generally covered by the intrinsically disordered regions, are suggested to play very important roles in driving liquid-liquid phase separation through Pi-Pi, hydrophobic, cation-Pi, and electrostatic interactions. These regions exhibit an overrepresentation of specific amino acid residues compared to the proteome proportion, such as the proline-arginine (PR)/glycine-arginine (GR) repeats, arginine-glycine-glycine (RGG) motifs, and prion-like domains [2].

The spike protein of the SARS-CoV-2 is an envelope glycoprotein that contributes mostly during the attachment process of the virus, its fusion, and host cell entry. It is also an important target for the development of vaccines, neutralizing antibodies, and inhibitors of viral entry. Its synthesis begins as a precursor protein which is cleaved into an amino-terminal S1 subunit composed of 700 amino acid residues and a carboxyl-terminal S2 subunit which is made up of 600 amino acid residues. Both residues respectively mediate the attachment and membrane fusion of the viral protein [3]. Our study is targeted at the design of a novel drug-like compound that can interfere with the molecular grammar that governs the liquid-liquid phase separation of the SARS-CoV-2 spike receptor-binding domain.

## Methods

**Fig. 1:**
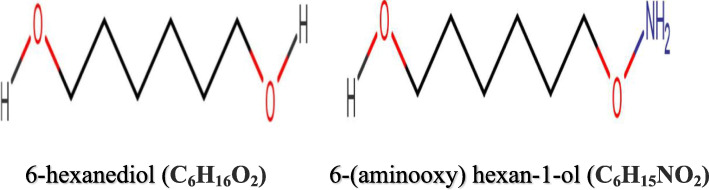
SARS-CoV-2 spike receptor-binding domain inhibitor design (2D structure)

Trivedi et al. [4] described the phase separation-driven inner centromere localization of the chromosomal passenger complex (CPC) by borealin, where the two regions making up the central disordered regions of the protein (as predicted by catGRANULE to display a high phase separation propensity) were deleted. The variant protein complex lacking this predicted borealin disordered region was deficient in both DNA-induced and spontaneous phase separation. We harnessed this approach in the prediction of the mechanism of action of our designed novel SARS-CoV-2 spike receptor-binding domain inhibitor. The inhibitor was designed through the structural modification of 1,6-hexanediol (Fig. 1), an aliphatic alcohol which is known for its ability to disrupt many phase-separated cellular organelles through the inhibition of weak hydrophobic interactions. Treatment of the chromosomal passenger complex with this alcohol reduced its enrichment in the inner centromere and likewise disrupted the in vitro coacervation of the ISB [4].

Waghorne et al. [5] described the design of novel chemotherapeutic agents (*N*,*N*-bis(glycityl)amines) that were prepared through the reductive amination of hexoses and pentoses and were subsequently screened for anti-cancer activity against cancerous virus. The amination approach was applied in the development of our novel inhibitor as shown in Fig. 1.

## Results and discussion

**Fig. 2:**
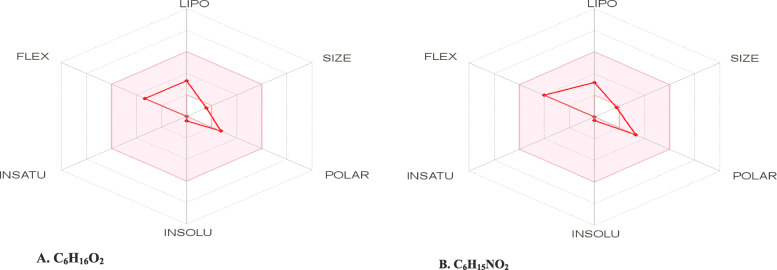
Bioavailability radar, showing the pharmacokinetic property summary of the experimental compounds

A comparative drug-likeness prediction test was carried out using in silico ADMET prediction tools (SwissADME and pkCSM) [6, 7]. The predictive outcome shows that both compounds share similar pharmacokinetic and toxicity properties (Tables 1 and 2; Fig. 2).

**Table 1:** Drug-likeness prediction

ADME parameters	C_**6**_H_**16**_O_**2**_	C_**6**_H_**15**_NO_**2**_
**Solubility**	Soluble	Soluble
**GI absorption**	High	High
**BBB permeant**	Yes	No
**P-gp substrate**	No	No
**Total clearance (log ml/min/kg)**	0.516	0.87

**Table 2:** Drug toxicity prediction

Toxicity parameters	C_**6**_H_**16**_O_**2**_	C_**6**_H_**15**_NO_**2**_
**Hepatotoxicity**	Inactive	Inactive
**Carcinogenicity**	Inactive	Inactive
**Cytotoxicity**	Inactive	Inactive
**Mutagenicity**	Inactive	Inactive

**Fig. 3:**
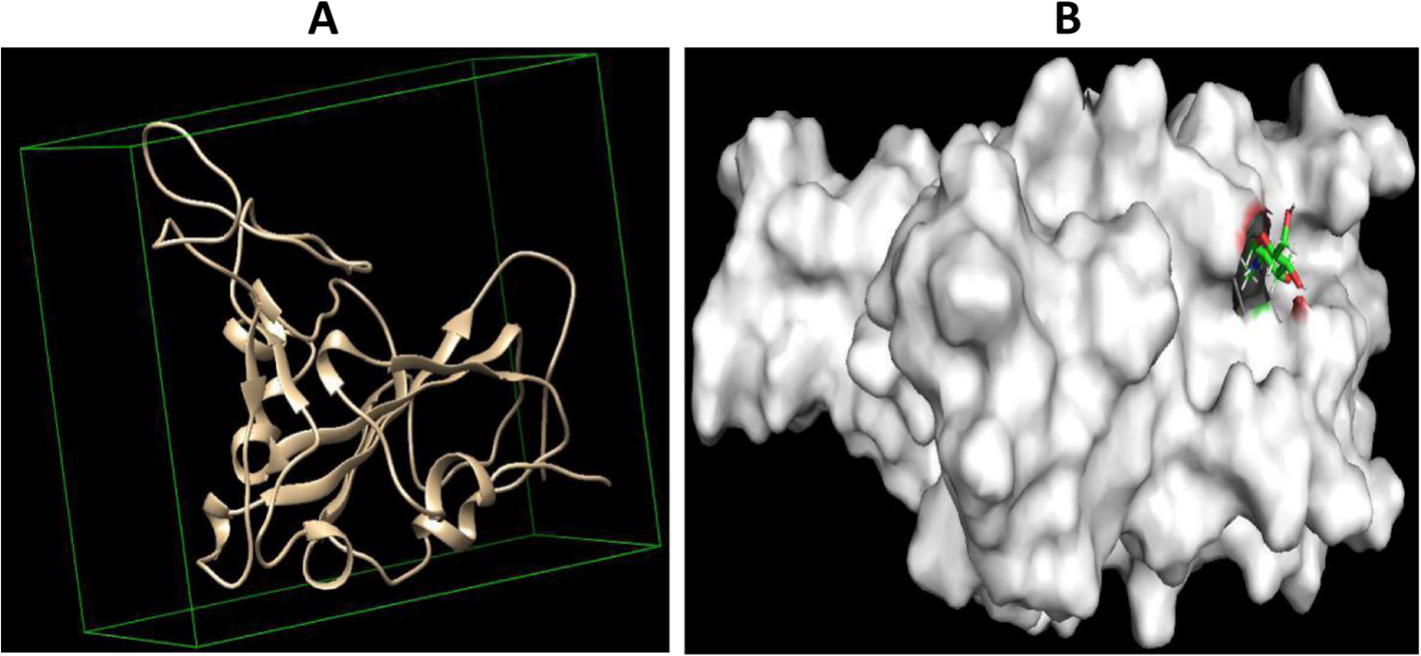
**a** The docking grid which was set to cover the whole surface of the viral protein (PDB: 6LZG). **b** The surface interaction view of the protein-drug complex

We directed a blind docking protocol towards the antiviral activity prediction of our novel compound. With this approach, the docked compound can interact with its most suitable region on the surface of the protein (Fig. 3). The interacting residues were then used in the prediction of the phase separation perturbation property of the compound.

**Fig. 4:**
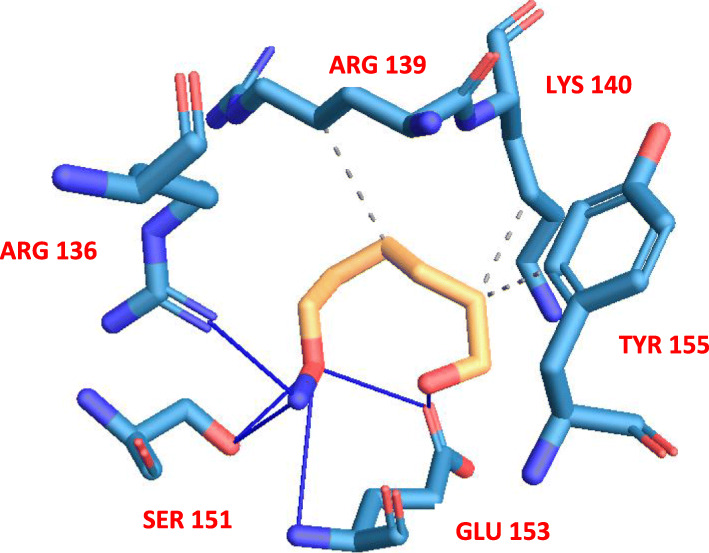
Inter-model interaction between bound drug and surface amino acid residues of the viral protein. Thick blue lines depict hydrogen bond interactions while gray broken lines depict hydrophobic interactions

As depicted in Fig. 4, five residues were involved in the inter-model interaction: hydrophobic interaction with lysine and tyrosine, and hydrogen bonding with serine and glutamate, while arginine was involved in both forms of interaction.

**Fig. 5:**
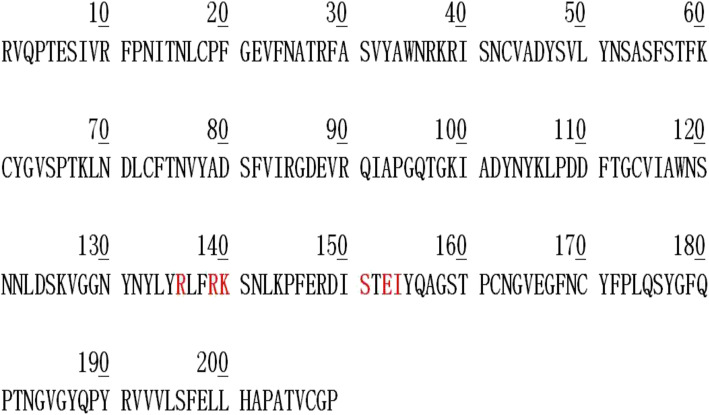
One-letter code amino acid sequence of the wild-type viral protein with the residues deleted to generate the variant, highlighted in red color

**Fig. 6:**
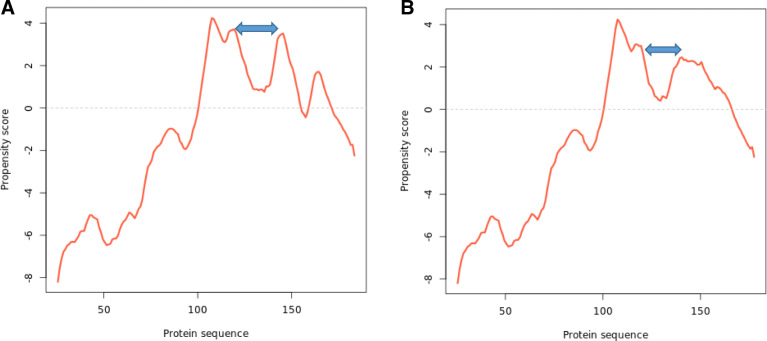
The liquid-liquid phase separation propensity profile plot of the wild-type and variant SARS-CoV-2 spike receptor-binding domain (**a** and **b**), respectively. The variant produced a reduced propensity score of 0.335 as against the propensity score of the wild type (0.388). The effect of the deleted residues on the propensity profiles of the two proteins is shown using arrows. The mutation caused a drastic reduction in the peaks of the variant (**b**) as compared to the wild-type protein (**a**). This shows the importance of the deleted residues in the liquid-liquid phase separation and functionality of the viral protein

The phase separation propensity of the wild-type SARS-CoV-2 spike receptor-binding domain was predicted using the catGRANULE algorithm [8] as described by Trivedi et al. [4]. Following the amino acid deletion approach as effected on borealin to give rise to a mutant, we generated a variant of the viral protein through the deletion of the interacting residues (Fig. 5) and subjected it to the catGRANULE algorithm. Results emanating from the predicted propensity score and profile showed that the drug interacted with important residues that drives the liquid-liquid phase separation, hence the functionality of the viral protein. The residues were also observed to fall within the intrinsically disordered region of the protein (Fig. 6).

Wang et al. [9] used an extensive mutagenesis approach in the identification of the sequence-encoded molecular grammar that underlies the driving forces of the FUS family proteins’ phase separation and concluded that it is preferentially governed by multivalent interactions among aromatic (tyrosine) and positively charged (arginine) residues. Based on the results from our in silico study, we hereby hypothesize that the molecular grammar governing the phase separation of the SARS-CoV-2 spike receptor-binding domain requires a more robust multivalent interaction, as predicted result revealed different residues with property variations (positively charged, negatively charged, non-charged, and aromatic residues), but common to all is their polarity.

## Conclusion

We have computationally identified a novel inhibitory drug-like compound (6-(aminooxy) hexan-1-ol) against the SARS-CoV-2 spike receptor-binding domain through its predicted interaction with the amino acid residues that drives the viral protein’s liquid-liquid phase separation. The compound was analyzed and predicted to possess satisfactory physiochemical, ADME, and toxicity properties. Findings from this study has shown that the compound has demonstrated drug-like attributes of being a potential inhibitor to be optimized for trial against the SARS-CoV-2 spike receptor-binding domain. We however recommend further experimental assessment of the antiviral potential of this compound.

## Data Availability

Not applicable.

## Abbreviations

ADME: Absorption-distribution-metabolism-excretion
ADMET: Absorption-distribution-metabolism-excretion-toxicity
BBB: Blood-brain barrier
CPC: Chromosomal passenger complex
DDX4: DEAD-Box 4
DNA: Deoxyribonucleic acid
GI: Gastrointestinal
IDRs: Intrinsically disordered regions
ISB: INCENP-survivin-borealin
LCRs: Low complexity regions
LLPS: Liquid-liquid phase separation
MLO: Membraneless organelle
PDB: Protein Data Bank
P-gp: P-glycoprotein
PR/GR: Proline-arginine/glycine-arginine
RGG: Arginine-glycine-glycine
RNA: Ribonucleic acid
SARS-CoV-2: Severe acute respiratory syndrome coronavirus 2
TDP-43: Transactive response DNA-binding protein-43
